# Hyperthermia Augments the H1-Histamine Receptor-Mediated Force in the Human Atrium

**DOI:** 10.3390/ijms26146842

**Published:** 2025-07-16

**Authors:** Thanh Hoai Pham, Peter Grundig, Britt Hofmann, Uwe Kirchhefer, Joachim Neumann, Ulrich Gergs

**Affiliations:** 1 Institute for Pharmacology and Toxicology, Medical Faculty, Martin Luther University Halle-Wittenberg, Magdeburger Straße 4, D-06097 Halle (Saale), Germany; hoai.pham@uk-halle.de (T.H.P.); peter.grundig@student.uni-halle.de (P.G.); ulrich.gergs@medizin.uni-halle.de (U.G.); 2 Department of Cardiac Surgery, Mid-German Heart Centre, University Hospital Halle, Ernst-Grube-Str. 40, D-06097 Halle (Saale), Germany; britt.hofmann@uk-halle.de; 3 Institute for Pharmacology and Toxicology, Medical Faculty, University of Münster, Domagkstr. 12, D-48149 Münster, Germany; kirchhef@uni-muenster.de

**Keywords:** histamine, histamine H_1_-receptor, hypothermia, hyperthermia, arrhythmia, inotropy, chronotropy

## Abstract

It was unknown whether hyperthermia increased the efficacy of histamine to raise the force of cardiac contractions via human H1-histamine receptors. To that end, we measured the force in isolated human atrial preparations (HAPs) excised from the right atrium of patients who underwent cardiac surgery due to severe two- or three-vessel coronary heart disease. For comparison, we also measured the force in paced (1 Hz) left and spontaneously beating right atrial preparations of transgenic mice overexpressing cardiac human H1-histamine receptors (H1-TG). Histamine (100 µM) was less efficient in raising the force in left atrial preparations from H1 TG mouse atria under hyperthermia than under hypothermia. Oppositely, histamine was more efficient in augmenting force during hyperthermia than during hypothermia in isolated electrically stimulated (1 Hz) HAPs. In sum, the contractile response to activation of H1-histamine receptor in H1-TG mice and in HAPs are opposite with regard to hyperthermia dependence. In patients with fever, histamine might thus be important, to preserve cardiac contractile function as a compensatory mechanism.

## 1. Introduction

Via its cardiac receptors, histamine augments the beating rate and raises the force of contraction in the hearts of humans. In HAPs, histamine also raised the first derivative of force versus time, a measure of innate contractility, and reduced the time needed for muscle relaxation, a measure of the function of the sarcoplasmic reticulum. The human heart contains four different histamine receptors. However, these contractile effects are presently thought to be mainly mediated by H_2_-histamine receptors and to a lesser extent via H_1_-histamine receptors.

**Figure 1:**
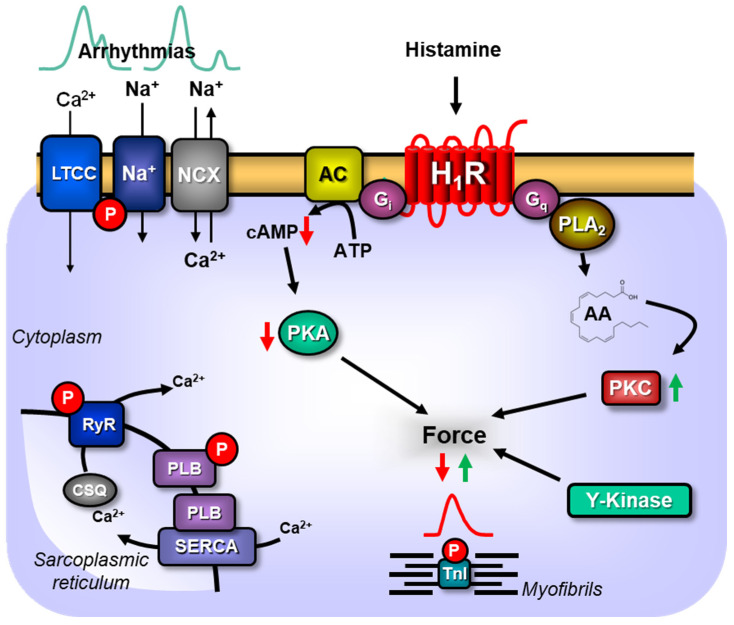
Scheme of the signal transduction of H_1_-histamine receptors in the atrium in cardiomyocytes. Histamine can stimulate H_1_-receptors in cardiomyocytes from transgenic mice overexpressing cardiac human H_1_-histamine receptors (H_1_-TG). In certain conditions the activity of adenylyl cyclase may be reduced. This leads to less formation of cAMP and less activity of the cAMP-dependent protein kinase (PKA). Thus, PKA is less able to phosphorylate and activate phospholamban (PLB), or the ryanodine receptor (RyR) or the L-type calcium ion channel (LTCC) or the sodium channel (Na^+^). Force may be increased by H_1_-histamine receptor (H_1_R) dependent stimulation of tyrosine kinase (Y-kinase) which can phosphorylate the myofibrils. This phosphorylation of myofibrils might increase their sensitivity for Ca^2+^ and thus more force is generated. Moreover, H_1_R may, via Gq, activate the phospholipase A2 (PLA_2_) leading to the formation of arachidonic acid (AA) which may activate the protein kinase C (PKC). PKC might also finally increase the force of contraction.

In the past, we studied transgenic mice with selective cardiac overexpression of the human H_2_-histamine receptor (H_2_-TG). In these H_2_-TG mice, histamine elevated atrial and ventricular contractility and intrinsic heart beat and led to tachycardia (Figure 1).

More recently, we established mice with selective cardiac overexpression of the human H_1_-histamine receptors (H_1_-TG, Figure 1). In these mice we reported biphasic inotropic effects of histamine: firstly, we observed a fall in the force of contraction and later and secondly a rise in contractility in the organ bath of the left atrium from H_1_-TG. Similarly, we recently noted that histamine increased the contractility of HAPs via H_1_-histamine receptors in the organ bath [1]. At present, it is unknown how H_1_-histamine receptor stimulation translates into a raise in force: a sensitization of myofilaments to Ca^2+^ and enhanced release of Ca^2+^ have been noted in left atrial preparations from guinea pig hearts, an accepted model for the cardiac H_1_-histamine receptor.

In this work, we wanted to find out how the function of the human H_1_-histamine receptor is changed in hyperthermia compared to hypothermia and normothermia (Figure 1). To this end, in the organ bath, we compared the response to temperature in HAPs and mouse atria during conditions that activate H_1_-histamine receptors.

Moreover, both hypothermia and hyperthermia, can induce atrial and/or ventricular arrhythmias [2,3,4]. Even when the heart is at normothermia, histamine can lead to atrial and/or ventricular arrhythmias, at least in isolated spontaneously beating right atrial preparations in H_2_-TG. Whether H_1_-histamine receptors cause arrhythmias also in hyperthermia and hypothermia is at present not known. In addition, there is incomplete knowledge about whether temperature can alter the positive inotropic potency or efficacy of histamine at high or low cardiac temperatures. Only some animal studies on this topic are available but studies in human cardiac tissue on the effect of histamine at different temperatures are non-existent. One may ask whether any change in the efficacy of histamine is clinically relevant. We argue here for the positive. Firstly, cardiac hypothermia is sometimes used during prolonged surgical procedures to reduce the metabolic demand to the heart. Secondly, cardiac hypothermia can occur via accident. For example, the British Royal Navy studied this topic: when navy sailors fled their sinking war boats in the North Sea in winter, some died from cardiac arrhythmia. Thirdly, cardiac bradycardia was noted in persons without shelter (homeless) in, e.g., Northern European cities in winter and mountain climbers in the snowy Alps. Hence, hypothermia is clinically relevant. Moreover, hyperthermia can occur in patients. Firstly, malignant hyperthermia is an accepted risk of narcosis using halothane or some muscle relaxants. Secondly, malignant neuroleptic syndrome (a rare but deadly side effect of antipsychotic drugs) can affect the temperature of the heart, leading to cardiac hyperthermia. Thirdly, sepsis is typically accompanied by cardiac hyperthermia. Nowadays, even sepsis has a poor prognosis in well-equipped hospitals where money is not an issue. Hence, cardiac hyperthermia is also a potential clinical problem.

Therefore, we studied mainly the hypothesis: hyperthermia increases the effect of H_1_-histamine receptor stimulation in HAPs. For comparison, and in order to be able to study arrhythmias, we also studied the right atrium of H_1_-TG.

## 2. Results

### 2.1. Mouse Atrial Preparations

**Figure 2:**
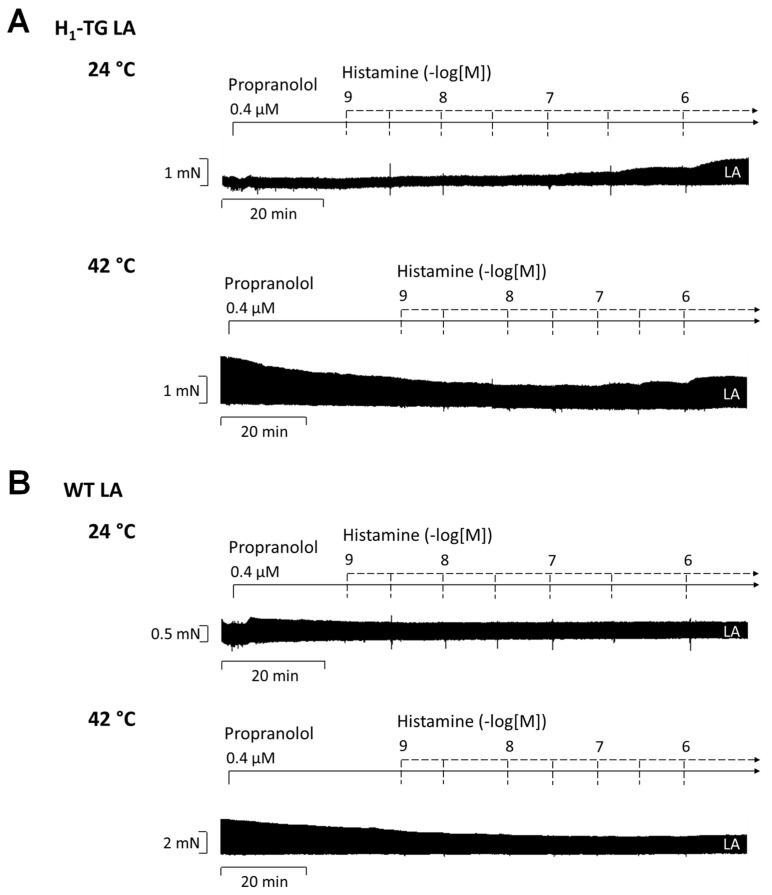

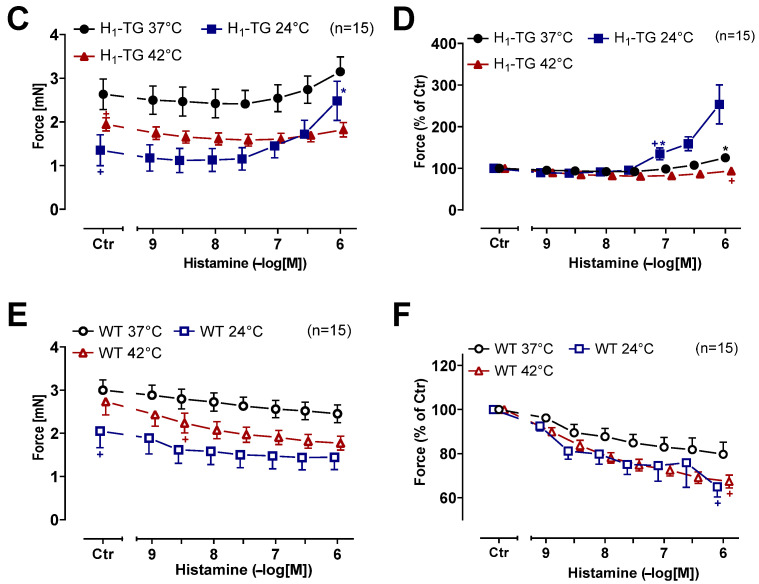
Histamine increases force of contraction in transgenic mice overexpressing cardiac human H_1_-histamine receptors (H_1_-TG) but not in wild-type (WT) littermates. Original recordings of the effect of increasing concentrations of histamine on electrically stimulated left atrial (LA) preparations from H_1_-TG (**A**) or WT (**B**) under hypothermia (24 °C) and hyperthermia (42 °C). The concentration-dependent effects of histamine on force of contraction in LA were summarized for H_1_-TG in absolute values (**C**) and in percent % (**D**), or for WT in absolute values (**E**) and in % ((**F**); WT 37 °C: test power (1 − β) = 0.646; WT 24 °C: test power (1 − β) = 0.295; WT 42 °C: test power (1 − β) = 0.958). In samples we added 0.4 µM propranolol to the organ bath in order to block β-adrenoceptors. Ordinates in (**A**–**C**,**E**) depict developed force of contraction in milli Newtons (mN). Abscissae indicate concentrations of histamine in negative decadic molar concentrations. Temperatures of the organ baths are indicated as circles (37 °C), squares (24 °C) or triangles (42 °C). “n” indicates the numbers of experiments. * First *p* < 0.05 vs. control (Ctr), ^+^ first *p* < 0.05 vs. 37 °C (One-Way ANOVA Bonferroni). Some error bars do not appear because they are shorter than the size of the symbols.

Basal parameters for mouse left and right atrial preparations were determined before drug application and are summarized in Table 1. The original tracings in Figure 2A for H_1_-TG and in Figure 2B for WT mice show a positive inotropic effect (PIE) of histamine. In the H_1_-TG mouse left atrial preparations, the histamine was more effective under hypothermia than under normothermia (Figure 2C). This effect is more easily demonstrated when the force of contraction is given as a % of control (Ctr) values, that is before relevant drug additions (Figure 2D). We also present how histamine changed contractility in atrial preparations from WT. Histamine failed to augment the force of contraction in the WT mice (Figure 2E,F).

**Figure 3:**
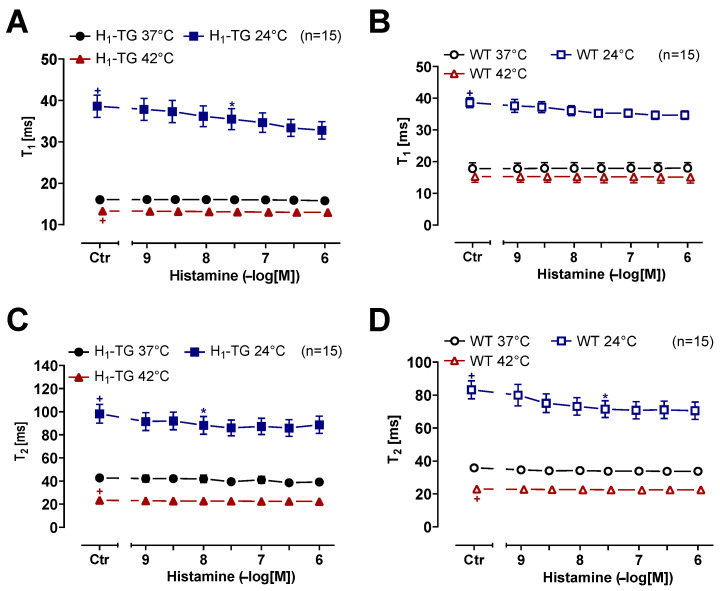
Histamine shortens time to peak tension under hypothermia in left atrium. Time to peak tension curve of transgenic mice overexpressing cardiac human H_1_-histamine receptors (H_1_-TG) (**A**) or wild-type (WT) (**B**) and time of relaxation curve of H_1_-TG (**C**) or WT (**D**) under normothermia (37 °C), hypothermia (24 °C) and hyperthermia (42 °C). We also added 0.4 µM propranolol to the organ bath in order to block β-adrenoceptors. Time to peak tension and time of relaxation under basal conditions (control: Ctr) were longer under hypothermia than under normothermia or hyperthermia in both left atrium from H_1_-TG (**A**,**C**) and WT (**B**,**D**). In addition, histamine shortens time to peak tension and time of relaxation in H_1_-TG under hypothermia only. Ordinates in (**A**,**B**) depict time to peak tension (T_1_) and in (**C**,**D**) time of relaxation (T_2_) in milli second (ms). Abscissae indicate concentrations of histamine in negative decadic molar concentrations. Temperatures of the organ baths are indicated as circles (37 °C), squares (24 °C) or triangles with tip pointing up (42 °C). “n” indicates the numbers of experiments. * first *p* < 0.05 vs. control (Ctr), ^+^ first *p* < 0.05 vs. 37 °C (One-Way ANOVA Bonferroni). Some error bars do not appear because they are shorter than the size of the symbols.

**Figure 4:**
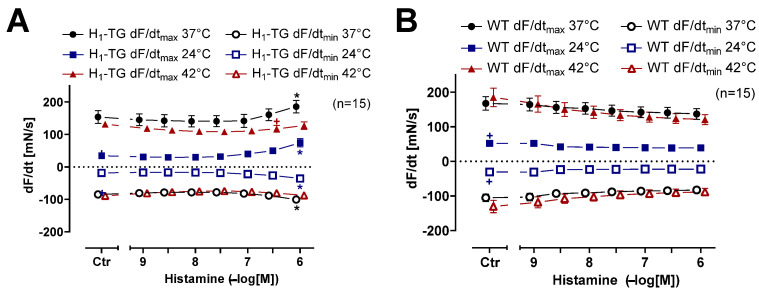
Histamine increases the rate of tension development and relaxation in left atrium in transgenic mice overexpressing cardiac human H_1_-histamine receptors (H_1_-TG). Concentration response curves for histamine in electrically stimulated mouse left atrium from H_1_-TG (**A**) and WT (**B**) under normothermia (37 °C), hypothermia (24 °C) and hyperthermia (42 °C). We added 0.4 µM propranolol to the organ bath in order to block β-adrenoceptors. Histamine induced an increase in the rate of tension development and relaxation in H_1_-TG mice under all thermal conditions. Ordinates in (**A**,**B**) depict rate of tension development (dF/dt_max_) and rate of tension relaxation (dF/dt_min_) in milli Newtons per second (mN/s). Abscissae indicate concentrations of histamine in negative decadic molar concentrations. Temperatures of the organ baths are indicated as circles (37 °C), squares (24 °C) or triangles with tip pointing up (42 °C). “n” indicates the numbers of experiments. * First *p* < 0.05 vs. control (Ctr), ^+^ first *p* < 0.05 vs. 37 °C (One-Way ANOVA Bonferroni). Some error bars do not appear because they are shorter than the size of the symbols.

Please note that hypothermia led to a longer time to peak tension (Ctr), compared to normothermia or hyperthermia, in left atrial preparations from H_1_-TG or WT mice (Figure 3A,B). Likewise, hypothermia prolonged the time of relaxation compared to normothermia or hyperthermia (Figure 3C,D). Histamine in H_1_-TG mice (Figure 4A) raised the maximum and minimum first temporal derivative force. Oppositely, histamine did not augment maximum and minimum first derivative of force of contraction in the WT mice (Figure 4B). In addition, histamine was less effective at augmenting the first derivative of force in H_1_-TG mice under hypothermia and hyperthermia than under normothermia (Figure 4A). In HAPs, during hypothermia histamine practical was without any inotropic effect. However, during hyperthermia histamine was more effective than in the presence of normothermia. In addition, the maximum and minimum first derivative of force in the atrium in WT mice were also lower under hypothermia than during normothermia and during hyperthermia, but were not changed with histamine (Figure 4B).

**Figure 5:**
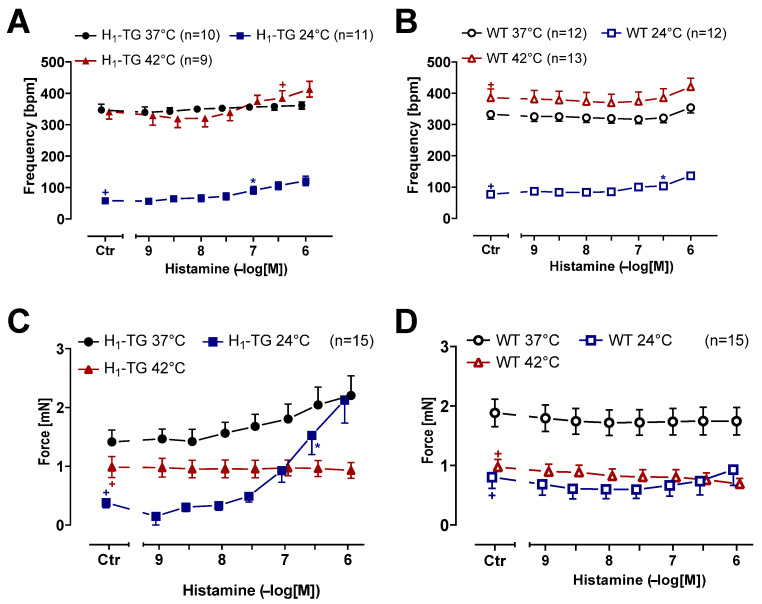
Histamine slightly increases the beating rate and the force of contraction in right atrium. Summarized concentration response curves for the effect of histamine on the beating rate in spontaneously beating mouse right atria under normothermia (37 °C), hypothermia (24 °C) and hyperthermia (42 °C) in transgenic mice overexpressing cardiac human H_1_-histamine receptors (H_1_-TG) (**A**) or wild-type (WT) (**B**) and on force of contraction in mN in H_1_-TG (**C**) or WT (**D**). We also added 0.4 µM propranolol to the organ bath in order to block β-adrenoceptors. Ordinates in (**A**,**B**) depict beats per minute (bpm), ordinates in (**C**,**D**) depict developed force of contraction in milli Newtons (mN). Abscissae indicate concentrations of histamine in negative decadic molar concentrations. Temperatures of the organ baths are indicated as circles (37 °C), squares (24 °C) or triangles with tip pointing up (42 °C). “n” indicates the numbers of experiments. * First *p* < 0.05 vs. control (Ctr), ^+^ first *p* < 0.05 vs. 37 °C (One-Way ANOVA Bonferroni). Some error bars do not appear because they are shorter than the size of the symbols.

**Figure 6:**
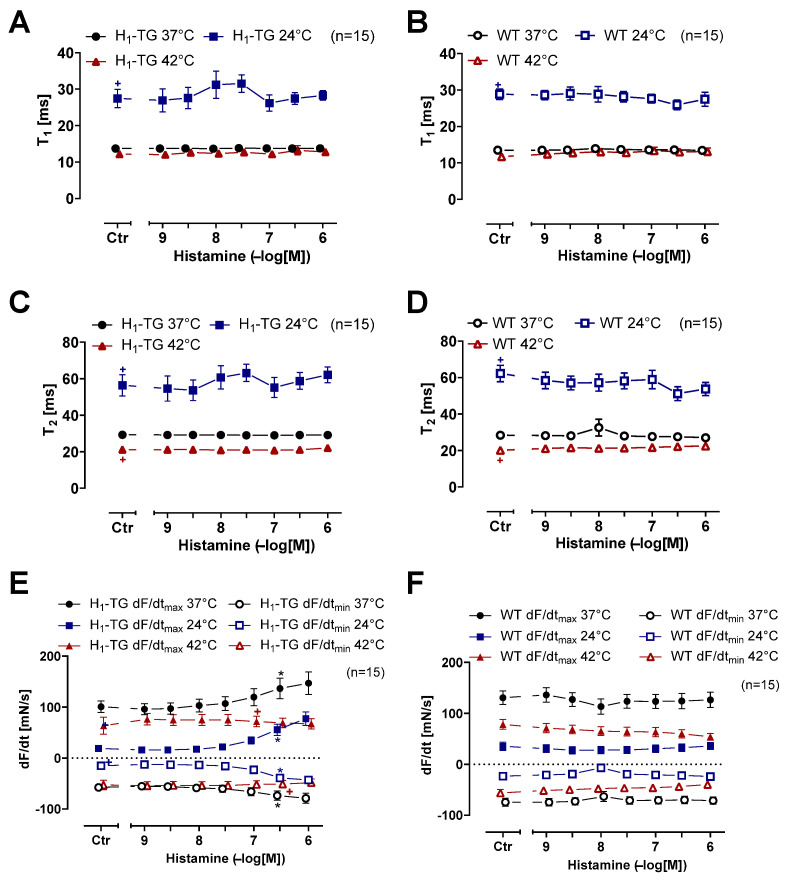
Hypothermia increases and hyperthermia decreases the effect of histamine on tension development and relaxation in RA. Concentration response curves for histamine in spontaneously beating RA from H1-TG and WT under normothermia (37 °C), hypothermia (24 °C) and hyperthermia (42 °C). We added 0.4 µM propranolol to the organ bath in order to block β-adrenoceptors. (**A**): Time to peak tension (T1) from H1-TG, (**B**): Time to peak tension (T1) from WT, (**C**): Time of relaxation (T2) from H1-TG, (**D**): Time of relaxation (T2) from WT, (**E**): Rate of contraction (dF/dtmax) and rate of relaxation (dF/dtmin) from H1-TG, (**F**): Rate of contraction (dF/dtmax) and rate of relaxation (dF/dtmin) from WT. Ordinates in (**A**,**B**) depict T1 in milli second (ms), (**C**,**D**) depict T2 in ms, and (**E**,**F**) depict dF/dtmax and dF/dtmin in milli Newtons per second (mN/s). Abscissae indicate concentrations of histamine in negative decadic molar concentrations. Temperatures of the organ baths are indicated as circles (37 °C), squares (24 °C) or triangles with tip pointing up (42 °C). “n” indicates the numbers of experiments. * First *p* < 0.05 vs. control (Ctr), + first *p* < 0.05 vs. 37 °C (One-Way ANOVA Bonferroni). Some error bars do not appear because they are shorter than the size of the symbols.

As a putative model for the human sinus node (which we could not obtain), we investigate the intrinsic frequency of the heart beat in isolated right atrial preparations. In unstimulated conditions (Ctr = before histamine was given), the beating rate was higher during hyperthermia and normothermia than during hypothermia in right atrial preparations from H_1_-TG and WT mice (Figure 5A,B). The histamine slightly raised the beating rate. Hence, frequency driven changes in beating rate due to histamine played a minor role. Therefore, we also decided to plot the force developed in the right atria of the H_1_-TG and WT mice (Figure 5C,D). As described before, histamine raised the force in the left atrium (Figure 2C) more markedly than in the right atrium (Figure 5C). During hypothermia, histamine shortened the time to peak tension (Figure 6A) and the time of relaxation (Figure 6C). Similar to the left atria (Figure 3C), the basal time of relaxation in the right atria under unstimulated conditions (Ctr) was higher at lower temperatures than at normal or higher temperatures (Figure 6C). In addition, as in the left atrial preparations (Figure 4A), histamine raised the first derivative of force in the right atrial preparations from the H_1_-TG mice during hypothermia as well as during normothermia (Figure 6E), but not in the right atrial preparations from the WT mice (Figure 6F).

**Figure 7:**
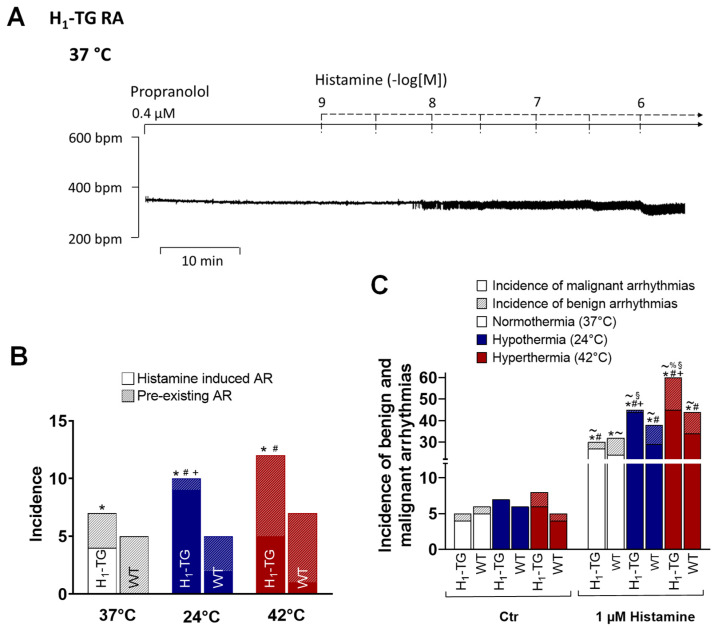
Histamine induces arrhythmias in transgenic mice overexpressing cardiac human H_1_-histamine receptors (H_1_-TG) and wild-type (WT) under all thermal conditions. Occurrence of arrhythmias at indicated temperatures in right atrium from H_1_-TG and WT. (**A**): Original recording of the effect of increasing concentrations of histamine on the beating rate in spontaneously beating mouse RA under normothermia (37 °C). We also added 0.4 µM propranolol to the organ bath in order to block β-adrenoceptors. Ordinate depicts bpm. Abscissa indicates concentrations of histamine in negative decadic molar concentrations. (**B**): Temperature-dependent incidences of arrhythmias during histamine addition in H_1_-TG and WT. Ordinate shows the incidences of the arrhythmias. Abscissa shows the different temperatures. * *p* < 0.05 vs. WT of histamine induced arrhythmias, ^+^ *p* < 0.05 vs. 37 °C of histamine induced arrhythmias (*χ*^2^-test), ^#^ *p* < 0.05 vs. 37 °C of total amount of arrhythmias (*χ*^2^-test). (**C**): Temperature-dependent average of incidences of benign and malignant arrhythmias before histamine application (CTR) and during maximal histamine addition (Max) in WT and H_1_-TG. Ordinate shows the incidences of the arrhythmias. Malignant arrhythmias: * *p* < 0.05 vs. CTR; ^+^ *p* < 0.05 vs. 37 °C, ^#^ *p* < 0.05 vs. WT (*χ*^2^-test). Benign arrhythmias: ^~^ *p* < 0.05 vs. CTR; ^%^ *p* < 0.05 vs. 37 °C, ^§^ *p* < 0.05 vs. WT (*χ*^2^-test). The number of experiments was n = 15.

As alluded to in the introduction, we were interested in whether arrhythmias and their incidences are altered by temperature in H_1_-TG mice. Data during normothermia are presented in Figure 7A, which shows that histamine induced arrhythmias. The results for incidences are plotted in bar diagrams in Figure 7B. Arrhythmias occurred more often under all thermic conditions in H_1_-TG mice than in WT mice (Figure 7B). To compare the types of arrhythmias, we have graphed the incidence of benign and malignant arrhythmias in another bar diagram (Figure 7C). One can see that in malignant arrhythmias were noticeably more often than benign ones and were observed more often in the H_1_-TG mice under hypo- and hyperthermia than in the WT mice (Figure 7C).

### 2.2. Human Atrial Preparations

**Figure 8:**
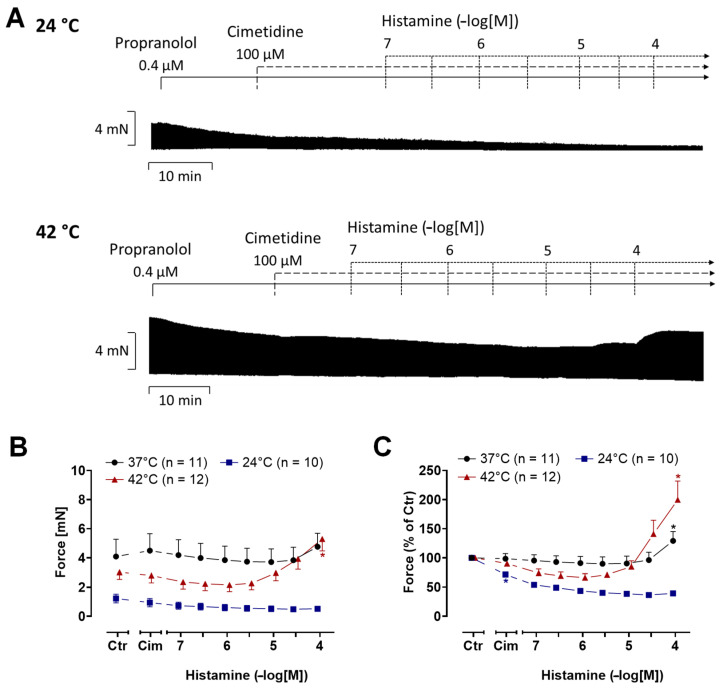
Histamine increases the force of contraction in human atrial preparations (HAPs) in a concentration-dependent manner. Original recordings of the effect of increasing concentrations of histamine in electrically stimulated (1 Hz) HAPs under hypothermia (24 °C) and hyperthermia (42 °C) (**A**). In addition, the concentration-dependent effects of histamine on the force of contraction were summarized in absolute values (**B**) and in % of pre-drug value (control: Ctr) (**C**). In the samples, we added 0.4 µM propranolol to the organ bath in order to block β-adrenoceptors as well as 100 µM cimetidine in order to block H_2_-histamine receptors. Histamine increases the force of contraction in HAPs under normo- and hyperthermia. The ordinates in (**A**,**B**) depict the developed force of contraction in milli Newtons (mN). Abscissae indicate concentrations of histamine in negative decadic molar concentrations. Temperatures of the organ baths are indicated as circles (37 °C), squares (24 °C) or triangles with tip pointing up (42 °C). “n” indicates the numbers of experiments. * First *p* < 0.05 vs. Ctr (One-Way ANOVA Bonferroni). Some error bars do not appear because they are shorter than the size of the symbols.

Basal parameters for human atrium were measured prior to drug administration and are presented in Table 2. In HAPs, histamine raised force in time- and concentration-dependent manner; this is evident in the original tracings in Figure 8A. The effect of histamine on force (measured in mN) are summarized in Figure 8B. The augmentation of force by histamine under normothermia or hyperthermia was not different from that during hypothermia (Figure 8C). The PIE of histamine reaches significance during normothermia and hyperthermia at higher histamine concentrations (from 3 µM to 100 µM for hyperthermia and from 10 µM to 100 µM for normothermia). Yet there was no PIE during hypothermia (Figure 8A–C). Histamine did not augment the contractile force during hypothermia.

**Figure 9:**
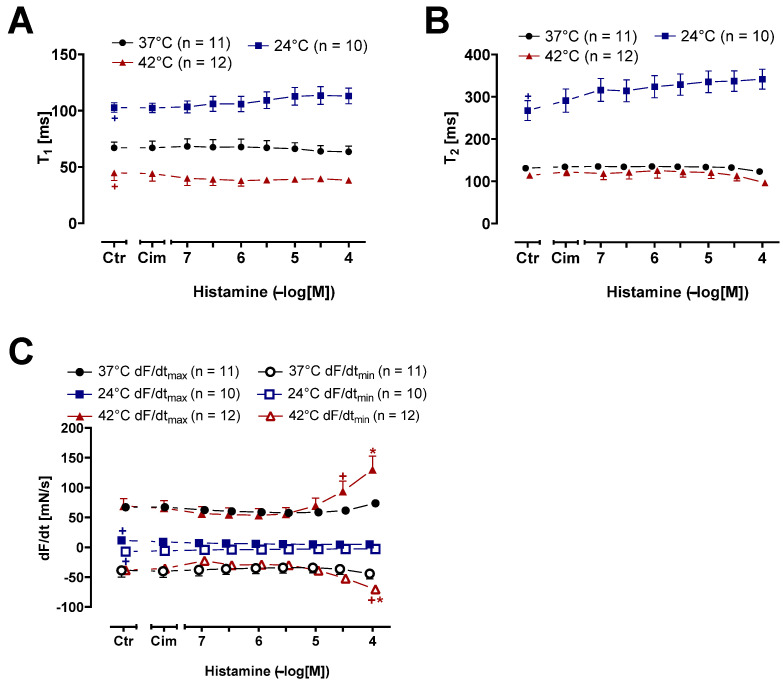
Histamine increases the rate of tension development and the rate of tension relaxation in human atrial preparations (HAPs). Summarized concentration response curves for histamine in electrically driven (1Hz) HAP. (**A**): Time to peak tension (T_1_ in milli second (ms)), (**B**): Relaxation time (T_2_ in ms); (**C**): rate of tension development dF/dt_max_ and rate of tension relaxation dF/dt_min_ (in milli Newtons per second (mN/s)). Abscissae indicate concentrations of histamine in negative decadic molar concentrations. Temperatures of the organ baths are indicated as squares (37 °C), circles (23 °C) or triangles with tip pointing up (42 °C). “n” indicates the numbers of experiments. * First *p* < 0.05 vs. control (Ctr); ^+^ first *p* < 0.05 vs. 37 °C (One-Way ANOVA Bonferroni). Some error bars do not appear because they are shorter than the size of the symbols.

In these HAPs (Figure 8), as in mice, we studied the contraction time and the relaxation time (Figure 9A,B). The time to peak tension under basal conditions, i.e., the pre-drug values (Ctr), lasted longer during hypothermia than during normothermia and hyperthermia (Figure 9A). Similarly, the basal time of relaxation (Ctr) was longer during hypothermia than under hyperthermia and normothermia (Figure 9B). In addition, histamine concentration-dependently increased the contraction time (Figure 9A) and relaxation time (Figure 9B) at histamine concentrations starting from 0.1 µM during hypothermia. During normothermia and hyperthermia, histamine shortened the time of relaxation at the highest histamine concentration (100 µM) (Figure 9B). We also plotted the maximum and minimum first derivative of the force of contraction (Figure 9C). Histamine raised the first temporal derivative of the force during normothermia, beginning at 10 µM. Histamine gave rise to higher values of the maximum and minimum first derivative of force. This started with 3 µM histamine under hyperthermia, whereas no changes were observed under hypothermia (Figure 9C).

## 3. Discussion

The main new finding, in our view, is the difference in temperature dependence between H_1_-histamine receptor and H_2_-histamine receptor stimulation in HAPs (Table 1 and Table 2). Namely, the efficacy of histamine to raise the force in HAPs at hypothermia was higher with H_1_-histamine receptor stimulation compared to normothermia (this report). In contrast, the efficacy of histamine to raise the force in HAPs at hypothermia was lower with H_2_-histamine receptor stimulation compared to normothermia [5]. Please note that in the present communication we included cimetidine in contractions in HAPs to exclude the stimulation of H_2_-histamine receptors in HAPs in contrast to our previous report [5].

We noted the same in left atrial preparations from H_1_-TG as in HAP: the efficacy of histamine to raise the force in left and right atrial preparations at hypothermia was higher with H_1_-histamine receptor stimulation compared to normothermia (this report). Likewise, the efficacy of histamine to raise force in left atrial preparations at hypothermia was higher with H_2_-histamine receptor stimulation compared to normothermia [5]. Hence, the HAPs and H_1_-TG behaved similarly, and the HAPs and H_2_-TG behaved oppositely in this aspect. We think this is an interesting new finding, because H_1_- and H_2_-histamine receptors use different signal transduction pathways. In more detail, it is generally accepted that the H_2_-histamine receptor exerts its positive inotropic and positive chronotropic effect via coupling of the H_2_-histamine receptor through stimulatory guanosine-triphosphate-binding proteins to adenylyl cyclase and an increase in cAMP in the cardiomyocyte. In contrast, the H_1_-histamine receptor does not use this pathway and does not increase cAMP levels in cardiomyocytes. At least in rabbits, H_1_-histamine receptor stimulation does not lead to an increase in inositol phosphates. In guinea pig left atrium (an often used model for the cardiac H_1_-histamine receptor), a reduction in potassium currents and/or increased phosphorylation of myofilaments have been convincingly shown by other groups. More specifically the phosphorylation of myofilaments occurred via the stimulation of tyrosine kinases and this tyrosine phosphorylation of myofilaments increase the Ca^2+^-sensitivity of the myofilaments [6]. We assume this mechanism is also active in the heart of H_1_-TG and more importantly the human heart. However, this is at present speculative and experiments in that direction are underway in our institution.

Of note, the contraction time and the relaxation time were prolonged in hypothermia in H_1_-TG and in WT. We saw a similar pattern in H_2_-TG and 5-HT_4_-TG before [5]. This prolonged contraction time and relaxation time were slightly reduced by histamine under hypothermia. This was unexpected because it did not occur under normothermia (or hyperthermia). Moreover, a shortening of the time to relaxation is typical of cAMP-increasing agents, and we have noted this in H_2_-TG and 5-HT_4_-TG before.

Hence, the mode of force generation via H_1_-histamine receptors appears to be altered in hypothermia. In normothermia, we failed to note a shortening of the relaxation time in H_1_-TG and in HAPs when we stimulated H_1_-histamine receptors. Another possibility is that the signficant prolongation of the time of relaxation under hypothermia simply unveils small effects that shorten the time to relaxation which are low and therefore too small to be detected under normothermia.

Before drug application, the derivative of force versus time was lower in hypothermia than normothermia in H_1_-TG and WT but also in the HAPs. This agrees with our findings in mouse cardiac appendages from WT and H_2_-TG or 5-HT_4_-TG in previous studies [5] and thus seems to be a general phenomenon.

Likewise, the basal frequency of the heart beat in H_1_-TG and WT in hypothermia is lower than in normothermia and this is consistent with our previous reports in H_2_-TG, 5-HT_4_-TG and their WT littermates [5].

The prolongation of contraction time and relaxation time, the lower force of contraction, and the lower rate of tension development in the right atrial preparations under basal conditions, which we report here for H1-TG and WT, are in agreement with our previous studies in H1-TG. Of note, we detected a similar pattern in HAPs with H1-histamine receptor stimulation.

Thus, we might hypothesize that the higher efficacy of H1-histamine receptors at low temperature might mean that, e.g., the Ca2+ sensitivity is more easily increased at lower than at higher temperature.

Now, we would like to compare our findings using histamine acting on H_1_-histamine receptor and on H_2_-histamine receptor with studies where cAMP was raised by the β-adrenoceptor. Isoprenaline directly stimulates cardiac β-adrenoceptors. In left atrial preparations from WT, isoproterenol exerted an inferior potency to augment the force of contraction at 24 °C than at 37 °C. Hence, one might come to the conclusion that a different mechanism must underlie the effect of histamine and isoprenaline on the force of contraction at lower temperature. In the isolated electrically paced left atrial preparations from guinea pigs, the positive inotropic effect of isoprenaline was shifted to the higher concentrations of isoprenaline at 25 °C compared to 37 °C preparation [7,8]. Hypothermia reduced the spontaneous beating rate. However, the positive chronotropic effect of isoprenaline exhibited a comparable potency at hypothermia and at normothermia in right atrial preparations from WT. Similarly, the intrinsic beating rate was less at 24 °C than at 37 °C but was augmented at hypothermia and normothermia in H_1_-TG when histamine was applied (Figure 5C).

Consistent with our present results, in left atrial preparations from guinea pigs (a model of the cardiac H_1_-histamine receptor) histamine was more effective in raising the force of contraction in hypothermia than in normothermia [7]. In contrast, in left atrial preparations from guinea pigs, the same preparations of isoprenaline were less effective in raising the force of contraction at hypothermia than at normothermia [7]. Hence, the temperature dependence of the efficacy of histamine to raise this force via the H_1_-histamine receptor is the same in guinea pig atria, the atria of H_1_-TG, and human atria, suggesting a similar biochemical alteration in the pathway for force generation.

Hyperthermia diminished force in isolated left atrial preparations and raised the intrinsic beating rate in right atrial preparations from H_1_-TG mice. The PIE of histamine showed reduced efficacy in H_1_-TG mice during hyperthermia. This is contrary to other reports in which histamine in guinea pig papillary muscles (via H_2_-histamine receptors) increased force less at hypothermia than at normothermia and in rabbit papillary muscles (via H_1_-histamine receptors) [8]. Hence, regional differences might exist in addition to species differences.

An enhanced incidence of arrhythmias is noted here for H_1_-TG compared to WT but was also detected for H_2_-TG. In contrast to the present findings, in H_2_-TG arrhythmias occurred more often in H_2_-TG than in WT during hyperthermia, but not during normothermia and hypothermia [5].

The mechanism of cardiac arrhythmia is probably different from that of H_2_-TG and 5-HT_4_-TG. This is likely, because the signal transduction is different. It is plausible that in H_2_-TG the arrhythmias are mainly caused by cAMP as probably all cAMP increasing agents increase the incidence of arrhythmias due to alterations in the Ca^2+^ homeostasis. This mechanism cannot involve cAMP in H_1_-TG because cAMP is not involved in the PIE of H_1_-histamine receptor stimulation. However, an alteration of Ca^2+^ homeostasis using other second messengers could well prevail. Other targets that may cause the arrhythmias in H_1_-TG might consist of potassium channels, an inhibition of sodium channel function and augmented currents through L-type Calcium currents or altered Ca^2+^-release from the sarcoplasmic reticulum [9,10,11,12,13,14].

### 3.1. Clinical Relevance

We have discussed the possible clinical relevance of cardiac H_1_-histamine receptors in depth elsewhere. Our data in right atrial preparations of H_1_-TG are, to the best of our knowledge, the first to indicate that H_1_-receptor stimulation can lead to atrial arrhythmias. Supraventricular arrhythmias are a well-known clinical problem. Our data suggest that in some patients these arrhythmias may be caused by an up-regulation of the H_1_-histamine receptor in the human atrium. Conversely, we can speculate that H_1_-histamine receptor antagonists might reduce these arrhythmias. However, caution is required as some antagonists like terfenadine inhibit potassium channels and thus induce arrhythmias. Hence, one might study better selective H_1_-histamine receptor antagonist lacking any action on ion currents or other receptors.

Likewise, our observation that H_1_-histamine receptor stimulation is more effective in HAPs in hyperthermia might have a clinical bearing. It is conceivable that in hyperthermia, H_1_-histamine receptor stimulation might be more relevant for contractile function in the human heart than H_2_-histamine receptor stimulation (cf. Table 3 for synopsis). This would have a beneficial positive inotropic effect and conceivably a detrimental proarrhythmic effect (Figure 7). Moreover, the cardiac effects of H_1_-histamine receptor antagonist may be more pronounced in patients suffering from hypothermia. However, this is also speculative.

### 3.2. Study Limitations

We have not addressed the signal transduction pathways of the H_1_-histamine receptor and how this is altered in hypothermia. To this end, one could measure tyrosine phosphorylation of myofilaments [15]. But that remains to be elucidated. Moreover, one can ask why we did not observe arrhythmias in HAPs. Indeed, the HAPs came from the right atrial appendages. However, they do not contain the human sinus node. Hence, in contrast to right atrial preparations, HAPs do not beat spontaneously. We always have to stimulate HAPs electrically. This may be compared to left atrial preparations which are also paced. In left atrial preparations from mice we do not see arrhythmias under the present experimental conditions. Hence, this electrically stimulation may explain why we never detected arrhythmias in HAP. Clearly, we also cannot rule out species differences.

We are well aware that several modes of normalization have been proposed in mouse and human atrial muscle preparations. In the past, the force was sometimes referred to the diameter of the muscle. Sometimes, one assumes a perfect circle for the section of the muscle and therefore one has recalculated the diameter of the muscle into a surface. However, over the years, we noted that this normalization did not reduce the scatter of data in mouse or human atrial preparations [15]. We have not presented EC50 values because the effects of histamine never reached a plateau which is a necessary condition to calculate valid EC50 values. Another limitation is the restricted availability of human atrial samples from patients who undergo cardiac surgery. This limitation inherently introduces a selection bias, as the majority of patients undergoing these procedures are male, resulting in a male-dominated dataset. Furthermore, ethical constraints preclude the collection of atrial tissue from individuals without cardiac pathology, thereby eliminating the possibility of including non-failing human controls. The limited sample size also impairs our ability to conduct statistically meaningful subgroup analyses, despite attempts to do so. Most likely, differences in signal transduction in mouse and human hearts must exist. These would explain why H_1_-histamine receptor stimulation have opposite temperature dependent inotropic effects in mouse atria compared to human atria. Future studies will focus on elucidating the role of tyrosine kinases in mediating inotropic responses, utilizing well-characterized enzyme inhibitors. We also aim to investigate the contribution of sarcolemmal potassium channels by applying selective pharmacological blockers, as previously demonstrated in non-human cardiac models by others (reviewed in [16]). Finally, due to the unavailability of atrial tissue from patients with persistent arrhythmias at our institution, we were unable to study arrhythmias in HAPs in the present study.

To sum up, there is a temperature dependence in the ability of histamine to raise force in H1-TG mice and of histamine in HAPs via H1-histamine receptors, and also in the histamine ability of histamine to raise the beating rate in H1-TG mice. However, during hyperthermia, atria from H1-TG and HAPs behave oppositely. Thus, different signal transduction mechanisms must be involved in human and mouse hearts, and these need to be elucidated.

## 4. Materials and Methods

### 4.1. Transgenic Mice

We have described the mouse model used previously: genetically modified mice, i.e., H_1_-TG mice. In H_1_-TG the human H_1_-histamine receptor is expressed with the help of the α-myosin heavy chain promoter. This promoter drives overexpression only in the heart and not, e.g., in the brain, where H_1_-histamine receptors are also well known. Moreover, this promoter does not drive expression in cardiac fibroblast, endothelial cells or smooth muscle cells. Mice were randomly chosen from both genders and were about 110 days old. We performed the keeping, raising and killing of the mice in accordance with the Animal Welfare Committee of our region. Permission had the number TS 10-24. The contraction conditions followed those used for mice and delineated in the preceding section.

### 4.2. Contractility in Isolated Mouse Atria

Mice were killed through cervical dislocation. The murine thorax was opened and the hearts were excised in toto [5]. The hearts were transferred to Petri dishes containing the modified Tyrode’s solution (vide infra) and further dissected under a microscope at ambient temperature. The left and right atrial appendages were carefully excised with a single scissor cut. Each appendage was very thin in diameter and weighed around 4 to 5 milligrams. The tissues were mounted vertically in 10 mL double-barreled organ baths, allowing precise control of bath temperature. Left atrial appendages and HAPs were electrically stimulated using rectangular pulses (5 V, 5 ms duration, 1 Hz) delivered via platinum electrodes connected to a Grass SD9 stimulator (Grass Instruments, Quincy, MA, USA). The organ baths were filled with modified Tyrode’s solution maintained at 37 °C using a Lauda M3 thermostat (Lauda, Königshofen, Germany) [17]. The solution was continuously gassed with a carbogen mixture (95% O_2_ and 5% CO_2_; Linde, Pullach, Germany) to maintain pH at 7.4. The composition of the modified Tyrode’s solution was as follows (in mM): 119.8 NaCl, 5.4 KCl, 1.8 CaCl_2_, 1.05 MgCl_2_, 0.42 NaH_2_PO_4_, 22.6 NaHCO_3_, 0.05 Na_2_EDTA, 0.28 ascorbic acid, and 5.05 glucose. Ascorbic acid is used here as an antioxidant. Isometric contractile force was recorded using a force transducer (Hellige, Freiburg, Germany), and signals were amplified via a Quad Bridge amplifier (ADInstruments, Oxford, UK), digitized using a PowerLab 8/38 data acquisition system (ADInstruments, Oxford, UK), and analyzed using LabChart 9 software (ADInstruments, Oxford, UK). In addition to recording the contractile force, the first derivative of force over time (dF/dt) was calculated. This parameter provides a more sensitive index of changes in myocardial contractility, particularly in response to pharmacological agents or thermal modulation [18]. Following recordings at normothermia (37 °C), the bath temperature was sequentially altered using digital temperature controls on the Lauda M3 thermostat (Königshofen, Germany). After initial measurements, the buffer was replaced three times and the temperature was reduced to induce hypothermia, after which a concentration–response curve for histamine was generated. Subsequently, the buffer was again exchanged, and the temperature was returned to 37 °C (normothermia) and finally heated to 42 °C to simulate hyperthermia, and a third concentration–response curve was obtained. Similar procedure has been used before in our studies [5]. In order to measure histamine H_1_-receptor-mediated inotropic effects, muscle strips were pre-incubated with 0.4 µM propranolol to antagonize β-adrenergic receptors, followed by 100 µM cimetidine to block histamine H_2_-receptors. Arrhythmias were classified into two categories: benign and malignant. In this report, benign arrhythmias were defined as contractions with varying amplitude, irregular inter-beat intervals, or isolated extrasystoles. Malignant arrhythmias are those arrhythmias which are not benign. This pragmatic classification system was adopted from our previous work to facilitate direct comparison with earlier findings in mouse atria and human atria studied under identical conditions [15].

### 4.3. Contractility in Isolated Human Atria

The tissue came from patients that underwent cardiac surgery. Patients received pre-medication and anesthesia was initiated. The thorax was opened. The right atrium was prepared by the surgeons. A catheter was inserted into the right atrium to start the extracardiac circulation. During this procedure a small piece of cardiac right atrial tissue was taken from the site of the needle insertion. The indications for surgery were two or three vessel coronary heart disease. Cardiac comorbidities included atrial fibrillation, heart failure, and hypertension. Cardiac drug therapy usually included bisoprolol (or a similar β-adrenoceptor antagonist), a loop diuretic like furosemide, an anticoagulant like apixaban, and acetyl salicylic acid as an antithrombotic agent. The human atrial samples were obtained from nine male patients, aged 58–85 years, and two female patients, aged 66 and 75 years. The tissue was transferred in a beaker containing the gassed modified Tyrode’s solution within thirty minutes by car to the pharmacological laboratory. Samples were cut at room temperature under a dissecting microscope. Like mouse atria, also HAPs were stimulated for 5 milliseconds. As in mice, the HAPs were also paced (1 Hz) electrically with platinum electrodes with rectangular impulses of direct currents from a Grass stimulator SD 9 (Quincy, MA, USA). Voltage ranged between five and ten Volts. As already described in the preceding section on mice, the output from the force transducer were moved into a bridge amplifier, digitized and kept on a computer (Dell, Halle [Saale], Germany) for further analysis. Again, the signals were measured using Lab Chart 8 (ADInstruments). The temperature changes and the contraction conditions followed those used for mice and delineated in the preceding section.

### 4.4. Data Analysis

Data are presented as the mean values ± standard error of the mean. Statistical significance was determined using an analysis of variance (One-Way ANOVA), which was followed by the Bonferroni *t*-test. More details can be found in the legends of the figures. We defined significance as a *p*-value smaller 0.05. Statistical analyses, including tests for normality, were conducted using GraphPad Prism 9.0 (GraphPad Software, San Diego, CA, USA). Effect sizes and statistical power have been calculated with G*Power 3.1 [19,20].

### 4.5. Drugs and Materials

We bought histamine dihydrochloride and propranolol hydrochloride from Merck, Dreieich, Germany. We further purchased chemicals only of the best purity that were commercially available. Deionized water was employed for the experiments. Stock solutions were dissolved daily.

## Figures and Tables

**Table 1:** Basal parameters (=before histamine addition) of mouse left and right atrial preparations.

Mouse left atrium				
basal tension [mN]	WT	3.15 ± 0.29 (n = 16)	2.16 ± 0.36 (n = 16)	2.73 ± 0.32 ^#^ (n = 16)
	H_1_-TG	2.63 ± 0.35 (n = 14)	1.35 ± 0.35 ^+^ (n = 14)	1.94 ± 0.15 (n = 14)
basal time-to-peak tension [ms]	WT	17.8 ± 1.92 (n = 16)	38.62 ± 1.67 (n = 16)	15.31 ± 2.01 (n = 16)
	H_1_-TG	16.04 ± 0.32 (n = 14)	38.6 ± 2.67 ^+^ (n = 14)	13.31 ± 0.26 ^+^ (n = 14)
basal relaxation time [ms]	WT	38.52 ± 1.85 ^#^ (n = 16)	83.21 ± 5.75 ^#^ (n = 16)	22.99 ± 1.61 (n = 16)
	H_1_-TG	42.7 ± 3.06 (n = 14)	98.18 ± 8.14 ^+^ (n = 14)	23.35 ± 0.91 ^+^ (n = 14)
Mouse right atrium		37 °C	24 °C	42 °C
basal tension [mN]	WT	1.88 ± 0.23 (n = 14)	0.8 ± 0.19 ^#^ (n = 14)	0.97 ± 0.13 (n = 14)
	H_1_-TG	1.41 ± 0.2 (n = 15)	0.38 ± 0.09 ^+^ (n = 12)	0.99 ± 0.18 (n = 14)
basal time-to-peak tension [ms]	WT	13.47 ± 0.21 (n = 14)	28.83 ± 1.47 (n = 14)	11.7 ± 0.31 (n = 14)
	H_1_-TG	13.71 ± 0.23 (n = 15)	24.93 ± 3.36 ^+^ (n = 15)	12.16 ± 0.32 (n = 15)
basal relaxation time [ms]	WT	28.34 ± 0.71 (n = 14)	62.21 ± 4.51 (n = 14)	20.06 ± 0.69 (n = 14)
	H_1_-TG	29.31 ± 1.23 (n = 15)	51.25 ± 7.34 ^+^ (n = 15)	21.16 ± 0.74 ^+^ (n = 15)
basal beating rate [bpm]	WT	274.03 ± 30.25 (n = 14)	170.6 ± 72.52 (n = 13)	344.15 ± 39.61 (n = 14)
	H_1_-TG	284.43 ± 31.55 (n = 14)	58.16 ± 10.45 ^+^ (n = 10)	348.21 ± 22.03 (n = 10)

H_1_-TG: left atrium of mouse with human H_1_-histamine receptor overexpression, WT: atrium from wild-type mouse, 37 °C: temperature in the organ bath= normothermia, 24 °C: temperature in the organ bath = hypothermia, 42 °C: temperature in the organ bath = hyperthermia. Number in brackets gives number of experiments. bpm: beats per minutes, ms: milliseconds, mN: milli Newton. Mouse right atrium: mouse right atrial preparations, mouse left atrium: mouse left atrial preparation. ^+^ *p* < 0.05 vs. 37 °C; ^#^ *p* < 0.05 vs. H_1_-TG.

**Table 2:** Basal parameters (=before histamine addition) of the human atrium.

Human Right Atrium	37 °C	24 °C	42 °C
basal tension [mN]	4.12 ± 1.13 (n = 11)	1.82 ± 0.5 ^+^ (n = 4)	3.69 ± 0.66 (n = 9)
basal time-to-peak tension [ms]	67.05 ± 5.88 (n = 11)	91.76 ± 6.37 ^+^ (n = 4)	47.95 ± 2.44 ^+^ (n = 9)
basal relaxation time [ms]	134.26 ± 4.54 (n = 11)	290.38 ± 11.48 ^+^ (n = 4)	103.74 ± 4.72 ^+^ (n = 9)

37 °C: temperature in the organ bath = normothermia, 24 °C: temperature in the organ bath = hypothermia, 42 °C: temperature in the organ bath = hyperthermia. Number in brackets gives number of experiments. mN: milli Newton; ms: milliseconds. Human atrium: HAP: human atrial preparation. ^+^ *p* < 0.05 vs. 37 °C.

**Table 3:** Opposite effects of histamine on force of contraction in paced atria of different species through different receptors.

Tissue	HAP: H_1_	HAP: H_2_	H_1_-TG	H_2_-TG
Hypothermia	No effect	Small PIE	PIE	Large PIE
Normothermia	Small PIE	PIE	PIE	PIE
Hyperthermia	Large PIE	Small PIE	No effect	Small PIE
Reference	This study	[5]	This study	[5]

HAP: human atrial preparations, HAP: H_1_: positive inotropic effect of H_1_- receptor stimulation in HAP, HAP: H_2_: positive inotropic effect of H_2_-receptor stimulation in HAP, H_1_-TG: left atrium of mouse with human H_1_-histamine receptor overexpression: H_2_-TG: left atrium of mouse with human H_2_-histamine receptor overexpression, PIE: positive inotropic effect.

## Data Availability

The data of this study are available from the corresponding author upon reasonable request.
